# A study of the changing characteristics and influencing factors of holiday visitor vitality in Urban parks: The case of Fuzhou, China

**DOI:** 10.1371/journal.pone.0311546

**Published:** 2024-12-05

**Authors:** Tingting Cui, Yongxiang Ye, Yingxin Zhuang, Qinlan Lin, Minlong Yan, Litian Zhang, Liying Zhu

**Affiliations:** Department of College of Landscape Architecture and Art, Master’s Degree Student, Fujian Agriculture and Forestry University, Fuzhou, China; Politecnico de Milano, SPAIN

## Abstract

The vitality of urban parks reflects the intensity of green space utilization, gauging visitors’ overall perception of the parks, facilitating integrated park management, and ensuring the parks’ sustainable development. But, the park’s spatial vitality characteristics change over time, and the factors influencing the differences in vitality have not been conclusively established. Therefore, This study employs Baidu heat map data to examine the spatial and temporal distribution patterns of park visitor vitality on holidays and weekdays in urban parks located in the core urban region of Fuzhou City. Meanwhile, this will be achieved by utilizing a geo-detector and MGWR model to examine the factors influencing visitor vitality and analyze the spatial variations in the impact coefficients. The conclusions are as follows: (1)Park vitality varied dramatically between different periods, with park vitality being higher on holidays than on weekdays. The peaks of vitality are all concentrated at 10:00 and 16:00. The park’s vitality on holidays had a pattern of many peaks, with a wave-like fluctuation. On weekdays, there was a notable M-shaped feature. (2)The spatial distribution of vitality has a "bimodal" pattern with two distinct cores and numerous fragmented fragments. There are notable variations in the spatial liveliness of different parks, characterized by a distinct "long-tail effect." In other words, there are just a few parks with high vitality, while many parks have low vitality. (3)The peripheral location features (G2) and the characteristics of transportation infrastructure (G3)are the main factors affecting park vitality; X11 amenities have the highest coefficient of impact on park vitality (0.501 on weekdays and 0.491 on holidays). The factors within the Park attributes (G1) and the park’s social media level (G4) showed a two-way interaction strength increase. (4)The coefficients of influence of impact factors on the space heterogeneity of vacation park vitality exhibit significant variation. The positive indicators have a spatial distribution that decreases from the northwest to the southeast, with the old city district having higher coefficients than the new city district. The negative indicators display the reverse pattern. This study offers scientific methodologies and recommendations for improving and designing urban park landscapes.

## Introduction

As the quality of life improves, citizens increasingly seek a lifestyle harmoniously linked with the natural ecological spatial environment. As public green space, urban parks are crucial components of the urban ecosystem, enhancing the city’s ecological landscape, fulfilling inhabitants’ outdoor sports requirements, and improving their quality of life and well-being [1–3]. Meanwhile, Urban parks serve as the primary physical environment where dwellers can connect with nature and engage in social activities. The activities of citizens within these parks reflect the quality and appeal of the physical environment [4,5], and the resulting pattern of spatial aggregation represents the park’s vitality. Park vitality is a metric that quantifies the amount of vitality and appeal of a park by measuring the frequency of visitor visits and the attractiveness of the park within a specific period [6]. Therefore, Accurate measurement of tourist visitation to urban parks is essential for effective park planning and management. It not only achieves the optimization of park landscape quality at microscopic level and improves the overall advantages of parks but also supports advancing urban ecological planning.

In conventional research, gathering data on the aggregation of park visitors’ activities primarily relies on on-site research, questionnaire surveys, and other expensive and labor-intensive approaches. However, the amount of data collected and the scope of the study could be relatively restricted. The emergence of geotagged social media enables the quantification of park visitor numbles in a significant and extended timeframe. Research has been carried out to measure park vitality utilizing various data sources such as Flickr, Twitter, cell phone signaling big data, Tencent travel big data, and so on [7–9]. The research primarily aims to quantify and forecast the level of vitality and liveliness exhibited by visitors to guide the effective management of scenic regions and urban planning and development [10–12], Pragya Bhatt used Flickr photographs to evaluate tourists’ perceptual representations of mapping in Chitwan National Park, located in Nepal [13], Yoonjung Kim conducts a cold hotspot aggregation study on tourist visits to ASEAN cultural places [14]. Nevertheless, the limitations of the conditions for utilizing social media data restrict the feasibility of conducting spatial research on a small scale. The Baidu Heat Map utilizes real-time crowd data monitoring features to measure spatial vitality at various scales [15]. Meanwhile, prior research has primarily examined quantitative assessments conducted over extended durations or simultaneously, disregarding variations in park vitality over time, particularly during certain holiday seasons.

Through extensive examination of spatial vitality, numerous scientists believe that the physical environment has the potential to influence spatial dynamism. Evaluating the vitality of public spaces for human activities has emerged as a significant research focus [14,16]. Prior research has confirmed the internal characteristics of parks [17,18], the surrounding environmental variables [19], and socio-economic factors [20]. According to previous studies, the internal characteristics of parks,the surrounding environmental variables, and socio-economic factors are the leading influencing indicators of the park’s vitality. In addition, other scholars have found that aspects such as park satisfaction on online platforms and park vitality also pass correlation tests. However, the specific ways these elements impact the vitality of the public spaces at different times, as well as the varying degrees of influence they have, remain unclear. Additionally, the coefficients of influence may change depending on the geographic location of the parks. Therefore, this study will analyze the impact indicators along different dimensions with park vitality. These studies contribute to the spatial planning of parks and the branding of resources to promote long-term stability in the vitality of the parks.

To summarize, while previous studies have utilized Baidu heat maps to measure the liveliness of public spaces, there are still areas for further investigation, which is the primary focus of this paper. One is the difference in spatial vitality characteristics of urban park vitality in different time periods, and the second is that the conclusions of different scholars’ influencing factors vary greatly in need of validation and discussion, and that the influencing factors play different roles in park vitality in different time periods. This study aims to quantitatively evaluate the differences and spatial distribution characteristics in visitor vitality between National Day holidays and weekdays in urban parks, taking 131 urban parks in the main urban area of Fuzhou. The study constructs influence indicators of different dimensions to explore the factors and spatial heterogeneity relationships that contribute to the differences in park vitality. This study aims to elucidate the preference traits of tourists, offer ways for enhancing the vitality of urban park visitors, and optimize the planned layout of urban parks. Ultimately, the goal is to maximize the utilization of urban park resources and facilitate sustainable urban growth.

## 2. Materials and methods

### 2.1 Research frameworks

**Fig 1:**
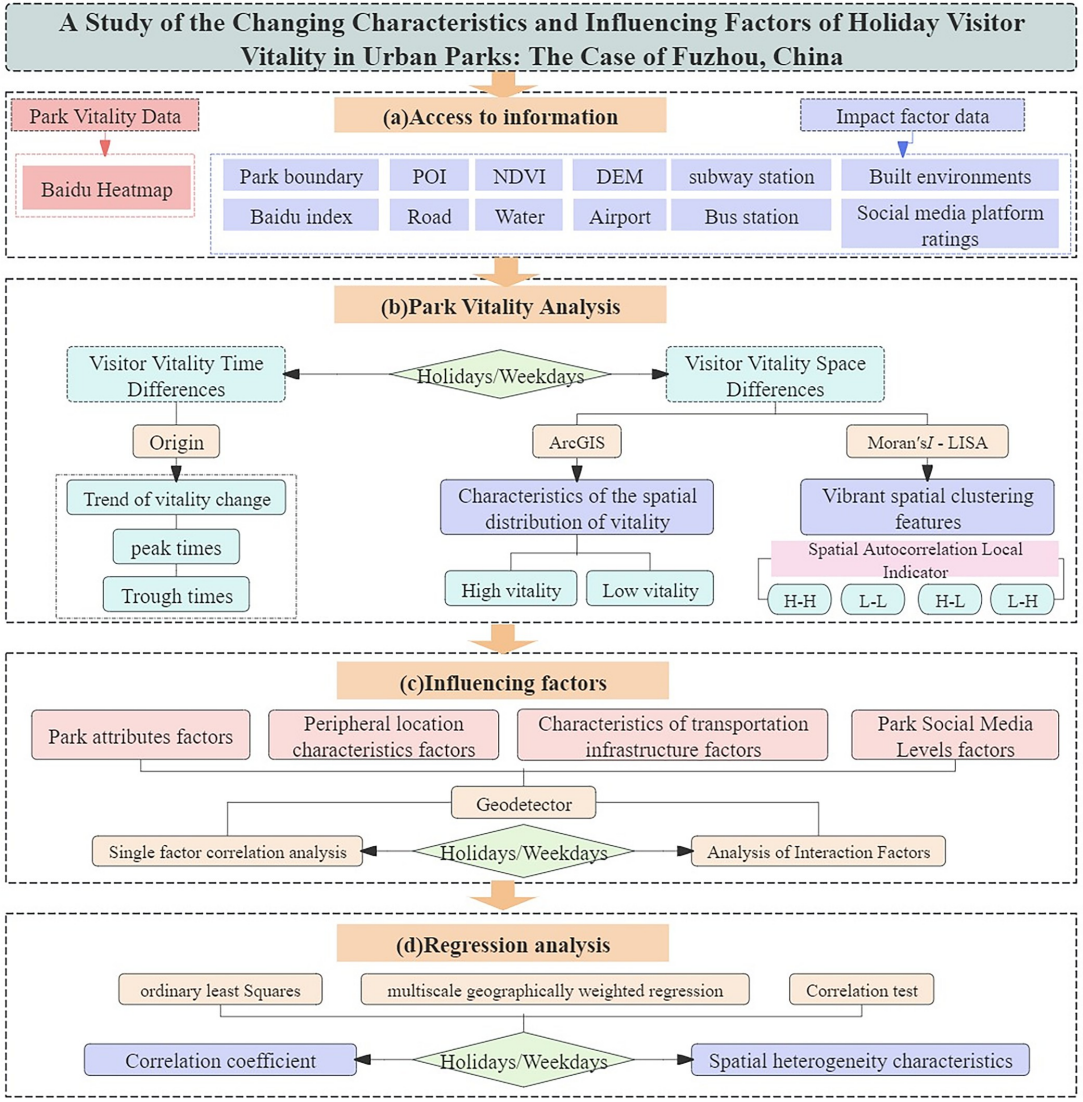
Research frameworks.

Fig 1 depicts the sequential steps involved in the complete analytical process. The primary objective is to thoroughly investigate the spatial distribution of visitor vitality in urban parks on holidays and weekdays, as well as the extent to which influencing factors and spatial heterogeneity play a role. This will enable us to understand the characteristics of vitality during different time periods and provide valuable insights for improving urban park planning. The research framework is structured into three components. Initially, we need to determine the Baidu thermal value of the parks throughout different periods. Subsequently, the spatial distribution of visitor vitality in several parks over various periods is charted using ArcGIS 10.7 software. Furthermore, Geodetector were employed to ascertain the factors that contribute to variations in park vitality, and the MGWR method was utilized to identify the components that contribute to variations in spaces heterogeneity in holiday park vitality.

### 2.2 Object of study

**Fig 2:**
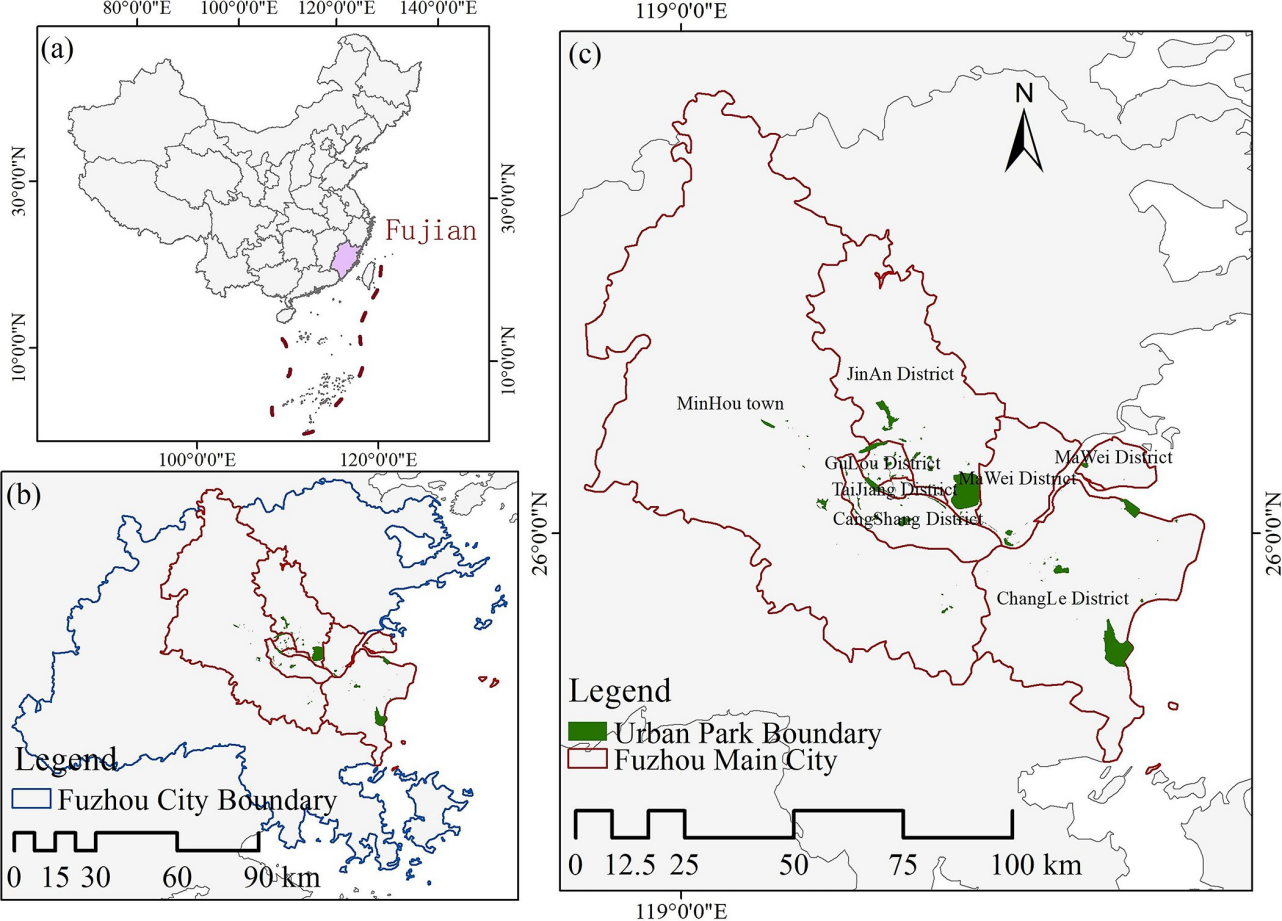
Overview of the study area.

Fuzhou (25°15′~26°39′N, 118°08′~120°31′E) is located at the eastern end of central Fujian Province in the southeastern coastal region of China (Fig 1A and 1B). It has a subtropical monsoon climate, warm and humid [21]. According to the 2023 Fuzhou City Statistical Bulletin the city’s parks and green areas amounted to 5,577.3 hectares, and the green space rate of the built-up area was about 40.3 percent, the per capita green park area will be 15m^2^. The ecological foundation of the area is favorable, making it a designated national riverside coastal ecological garden city. The city exercises control over 6 municipal districts, 6 counties, and 1 county-level city [22]. Gulou District, Cangshan District, Taijiang District, and Jin’an District are part of the old city district, whilst Minhou County, Changle District, and Mawei District constitute the new city district. We utilized geospatial data from the Fuzhou City Park List and Gaode Map’s online open platform to analyze 208 parks [23]. After excluding parks without geographic information boundaries, we identified 131 urban parks in the main urban area of Fuzhou for our research (Fig 2C).

### 2.3 Data sources and preprocessing

#### 2.3.1 Park vitality data

**Fig 3:**
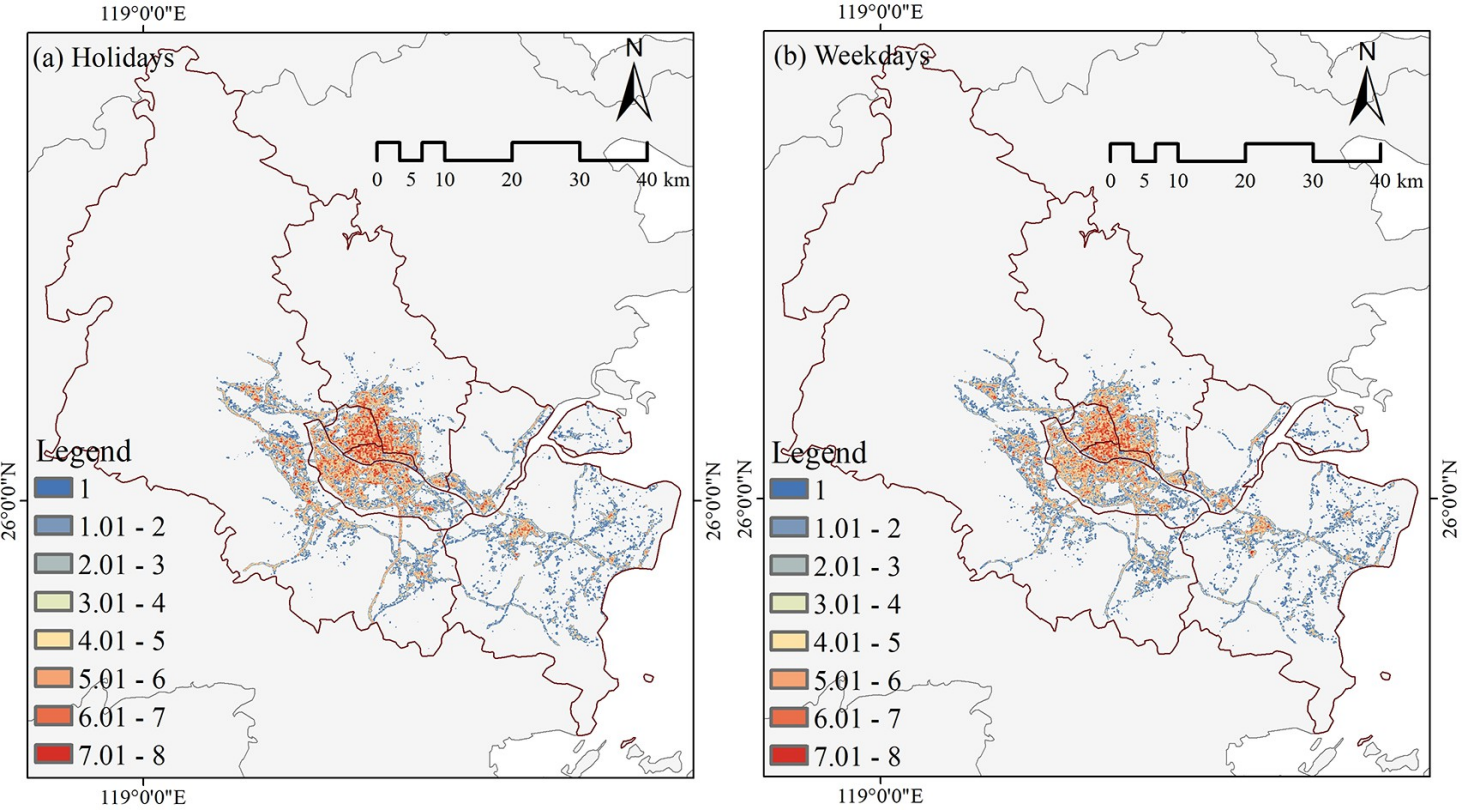
Baidu heat map average.

This research measured the level of visitor vitality in each park using a Baidu heat map collected for the National Day Holiday 2022 (October 1, 2022—October 7, 2022) and one working week in 2023 (October 30, 2023—November 3, 2023). The data was obtained using timed tracking from 7:00 to 22:00, with a time interval of 1 hour. The Baidu heat map has an accuracy of 17 levels, and 192 raster data were obtained. Afterward, we use the reclassification tool in ArcGIS10.7 to recognize some areas in the park, such as dense forests and bodies of water, that are inaccessible to tourists. These areas vitality values of "0" will be reassigned to "NODATA." Additionally, the raster calculator and the mask extraction tool were employed to obtain the visitor vitality measurements for 131 urban parks. The Baidu Heat Map displays the average visitor vitality during the National Day holidays and regular work weeks on holidays (Fig 3A) and weekdays (Fig 3B).

#### 2.3.2 Park visitor vitality impact indicators data

The quantitative analysis of the influencing factors, this study mainly selected 25 influencing indicators in 4 dimensions: the park’s characteristics, the characteristics of the surrounding area, the characteristics of the transportation environment, and the level of the park’s social media (S1 File). Among them. The park’s characteristics, G1, are an essential material basis for meeting tourism needs. Area, water body, NDVI, DEM, and SLOPE are the primary factors to measure the park’s natural environment characteristics. Still, the park’s history of establishment, internal facilities, and road density reflect the critical indicators of the park’s infrastructure improvement and cultural resource richness. Surrounding location characteristics (G2) and transportation environment and facilities characteristics (G3) reflect the accessibility of the infrastructure around the park’s exterior, which directly affects the arrangement of tour schedules and the satisfaction of tourists’ travel experience. Park social media levels (G4) reflect an externalized tool for park influence and popularity. Therefore, this study analyzes the relationship with park vitality in the four dimensions. All data sources and collection times are shown in (Table 1).

**Table 1:** Data source.

Type	Variant	Variant Description	sources
**Park District Data**	City parks and green spaces		https://ylj.fuzhou.gov.cn/, accessed on 17 February 2022; https://lbs.amap.com/, accessed on 1 October 2022;
	Administrative boundaries		https://www.resdc.cn/, accessed on 1 October 2022
**Park Vitality Data**	Vitality data		https://map.baidu.com/, accessed on 1–7 December 2022 and 30 December—3 November 2023
**Park attributes (G1)**	Park area (X1)	Area within the geographic boundaries of the park	https://lbs.amap.com/, accessed on 18 October 2022
	Water body area (X2)	Modified normalized difference water index(MNDWI)/area of the park	earthexplorer.usgs.gov; https://developers.google.cn/earth-engine/, accessed on 15 October 2022
	Green Coverage (X3)	Normalized differnce vegetation index (NDVI)/area of the park	https://www.resdc.cn/, accessed on 18 October 2022
	Internal facilities (X4)	Number of POIs within the park/area of the park	https://lbs.amap.com/, accessed on 18 October 2022
	Internal road network density (X5)	Length of road network within the park/area of the park	https://www.openstreetmap.org/,accessed on 15 October 2022
	Park building history score (X6)	The history of the founding of the park is noted as "1" for >500, "0.8" for >100, "0.6" for 50–100, " 0.4", 0–20 "0.2"	https://ylj.fuzhou.gov.cn/, accessed on 15 December 2023
	Average elevation (X7)	Average elevation within the park boundaries	https://www.resdc.cn/, accessed on 15 October 2022
	Flat slope gradient (X8)	Gradient of flat slopes within park boundaries	https://www.resdc.cn/, accessed on 15 October 2022
**Peripheral location characteristics (G2)**	Business Office Facilities (X9)	Business office POI density within 500m from the park/area of the park	https://lbs.amap.com/, accessed on 15 October 2022
	Education and Medical Facilities (X10)	Education and Medical POI density within 500m from the park/area of the park	https://lbs.amap.com/, accessed on 15 October 2022
	Living Service Facilities (X11)	Living Service POI density within 500m from the park/area of the park	https://lbs.amap.com/, accessed on 15 October 2022
	Recreational Facilities (X12)	Recreational POI density within 500m from the park/area of the park	https://lbs.amap.com/, accessed on 15 October 2022
	Building Density (X13)	Building density within 500m from the park/area of the park	https://www.openstreetmap.org/,accessed on 15 October 2022
	Average building height (X14)	Average building height within 500m from the park/area of the park	https://www.resdc.cn/, accessed on 1 June 2023
	Residential accommodation facilities (X15)	Density of Residential accommodations within 500m from the park/area of the park	https://lbs.amap.com/, accessed on 15 October 2022
	Commercial shopping facilities (X16)	Density of commercial shopping facilities within 500m from the park/area of the park	https://lbs.amap.com/, accessed on 15 October 2022
	Number of parking lots (X17)	Density of Number of parking lots within 500m from the park/area of the park	https://lbs.amap.com/, accessed on 15 October 2022
**transportation infrastructure (G3)**	Road network density (X18)	Density of road network within 500m from the park	https://www.openstreetmap.org/,accessed on 15 October 2022
	Average travel time cost (X19)	"Small-o Maps" calculation acquisition	"Small-o Maps" APP, accessed on 15 October 2022
	Bus stop density (X20)	Density of bus stops within 500m from the park	https://lbs.amap.com/, accessed on 15 October 2022
	Density of subway stations (X21)	Density of subway entrances within 500m from the park/area of the park	https://lbs.amap.com/, accessed on 15 October 2022
	Park to Airport Time (X22)	"Small-o Maps" calculation acquisition	"Small-o Maps" APP, accessed on 15 October 2022
	Park to Fuzhou Station Time (X23)	"Small-o Maps" calculation acquisition	"Small-o Maps" APP, accessed on 15 October 2022
	Park to Fuzhou South Station Time (X24)	"Small-o Maps" calculation acquisition	"Small-o Maps" APP, accessed on 15 October 2022
**the park’s social media level(G4)**	Park network attention (X25)	Average park score comparison between holidays and weekdays	https://index.baidu.com/; https://www.ctrip.com/; https://www.dianping.com/; ttps://ditu.amap.com/; https://www.mafengwo.cn/?city8.com; "WeChat-index"APP, accessed on 15 October 2022

### 2.4 Research methods

#### 2.4.1 Spatial visualization of parks

This study summarizes the Baidu heat map measurements within a 500-meter radius of the park and calculates the average value of park vitality throughout different time periods. In addition, spatial visualization of park vitality at different levels was performed using the Natural Break (Jenks) method in the ArcGIS 10.7 program.

#### 2.4.2 spatial autocorrelation

A global spatial autocorrelation analysis Moran’s *I* index measures the degree of spatial resemblance between neighbouring or adjacent locations. It aids in determining the clustering and scattering of the spatial arrangement of park vitality [24]. The calculation of the worldwide Moran’s *I* index is performed as follows:

$$I=(\sum_{i=1}^{n}\sum_{j=1}^{m}W_{ij}(X_{i}-\bar{X})(X_{j}-\bar{X}))/((\sum_{i=1}^{n}\sum_{j=1}^{m}W_{ij}(X_{i}-\bar{X}))\times (\sum_{i=1}^{n}\sum_{j=1}^{m}W_{ij}))$$ (1)

*n* is the number of calculation units, *m* is the number of neighboring units of a calculation unit, *X*_*i*_ is the value of unit i, *X*_*j*_ is the value of unit j in the neighborhood of unit *i*, $\bar{X}$ is the average of all cell values, *W*_*ij*_ is a spatial adjacency matrix, the range of results for the calculation of the *I* index is [–1, 1], a value greater than 0 shows a positive spatial correlation, whereas a negative value indicates a negative correlation. A value equal to 0 means the lack of geographical correlation.

The Local Indicators of Spatial Association (LISA) method is employed to determine the level of correlation between a specific spatial element and its nearby elements. It also helps identify whether spatial elements are clustered together or dispersed on a local level [25,26]. We utilize it to precisely delineate the spatial clusters of vitality within particular regional parks. The Moran’s I index is computed in a confined manner:

$$I=Z_{i}\sum_{i}W_{ij}Z_{j}$$ (2)

*Z*_*i*_ and *Z*_*j*_ Standardized variables refer to variables that have been transformed to have a mean of zero and a standard deviation of one, *Z*_*i*_ *= (X*_*i*_ - $\bar{X}$) / *δ, δ* is the standard deviation of *X*_*i*_.

Elevated values of local Moran’s *I* demonstrate the presence of spatial clustering among area cells that have comparable variable values, and conversely, the presence of geographical clustering among space cells that have distinct values. By comparing the amount of the Moran’s *I* index across parks and surrounding regional parks, we can categorize park spatial correlations into five aggregation types: high-high, low-low, low-high, high-low, or unimportant. The terms "high-high" and "low-low" describe the similarity in vitality levels between the park and its nearby parks, where both are either higher or lower in vitality. Similarly, "high-low" and "low-high" allude to situations where external forces might create anomalies in vitality levels.

#### 2.4.3 Geodetector

Geodetector are a statistical technique used to identify the geographical differences between geographic features and uncover the underlying factors that influence them [27–30]. This study uses probes to identify components and analyze their influence by quantifying the number of their forces through an equation:

$$q=1-\frac{\sum_{h=1}^{L}N_{h}\sigma_{h}^{2}}{N\sigma^{2}}$$ (3)

*q* represents the extent to which the factors influencing the vitality and liveliness of visitors in urban parks are clarified; *L* is the stratification of the dependent or independent variables; *N*_*h*_ and $\sigma_{h}^{2}$ are the number of classifications and variance of layer *h*, respectively; *N* and *σ*^*2*^ represent the total number of classifications and the variation for the entire research area, respectively. The range of values of *q* is 0–1, A higher *q*-value implies a driver that has a more significant impact on visitor vitality in urban parks, whereas a lower *q*-value suggests a weaker influence.

Interaction is employed to examine the relationship between several factors and determine if the combined effect of two or more factors can enhance or diminish the explanatory power of the dependent variable, represented as *q*(A∩B). This elucidates the intricate interplay among several factors that influence alterations in park vitality. There are five distinct categories of factor interaction types, specifically, the impacts of interactions on vigor (Table 2).

**Table 2:** Types of interaction between two factors.

Criterion	Interaction
*q(X1∩X2)<Min(q(X1)*,*q(X2))*	Nonlinear weakening
*Min(q(X1)*,*q(X2)) < q(X1∩X2)< Max(q(X1)*,*q(X2))*	Single-factor nonlinear attenuation
*q(X1∩X2)> Max(q(X1)*,*q(X2))*	Two-factor enhancement
*q(X1∩X2) = q(X1)+q(X2)*	Independent
*q(X1∩X2)>q(X1)+q(X2)*	Nonlinear enhancement

#### 2.4.4 Regression modeling analysis

Ordinary least squares (OLS) models are employed to examine global regression models that analyze the connection between influencing factors and the intensity of park visitor visitation. These models also enable the assessment of covariance between influencing factors and the removal of elements that do not pass covariance [31]. The principle of this model is expressed as follows:

$$y_{i}=\beta_{0}+\sum_{k=1}^{m}\beta_{k}x_{ik}+\varepsilon_{i}$$ (4)

*y*_*i*_ represents the the spatial *i* location dependent variable. *β*_*0*_ is a constant term, while *β*_*k*_ represents the regression coefficient of the *k*th independent variable. The value of the *i*th independent variable at location *k*th in space is denoted as *x*_*ik*_, and *ε*_*i*_ represents the random error term.

The Multi-scale Geographically Weighted Regression can analyze the spatial characteristics of the regression parameters and examine the spatial heterogeneity of the factors influencing the spatial distribution of park vitalit [32,33]. The model is derived from the standard GWR model and addresses the limitations of bandwidth selection. This allows for the selection of different bandwidth values for distinct variables, so capturing the spatial heterogeneity among variables more effectively and enhancing the accuracy of regression analysis. the principle can be expressed as follows:

$$Y_{i}=\beta_{\mathrm{bwo}}(u_{i}+v_{i})\sum_{j=1}^{m}\beta_{\mathrm{bwj}}(u_{i},\;v_{i})x_{ij}+\varepsilon_{i}$$ (5)

where *yi* represents the vitality of the *i*th unit, *x*_*ij*_ represents the evaluation index of the *j*th factor of unit *i*, *m* is the number of independent variables included in the local model of the *i*th unit, *ε*_*i*_ is the random error term of the ith unit; *β*_bwj_ (u_*i*_, v_*i*_) represents the local regression coefficient of the *j*th factor of unit *i*; *bwj* is the optimal bandwidth of the *j*th factor; (u_*i*_, v_*i*_) is the position of the *i*th unit, and *β*_bwo_ is the intercept under optimal bandwidth.

## 3 Results

### 3.1 Visitor vitality time differences

**Fig 4:**
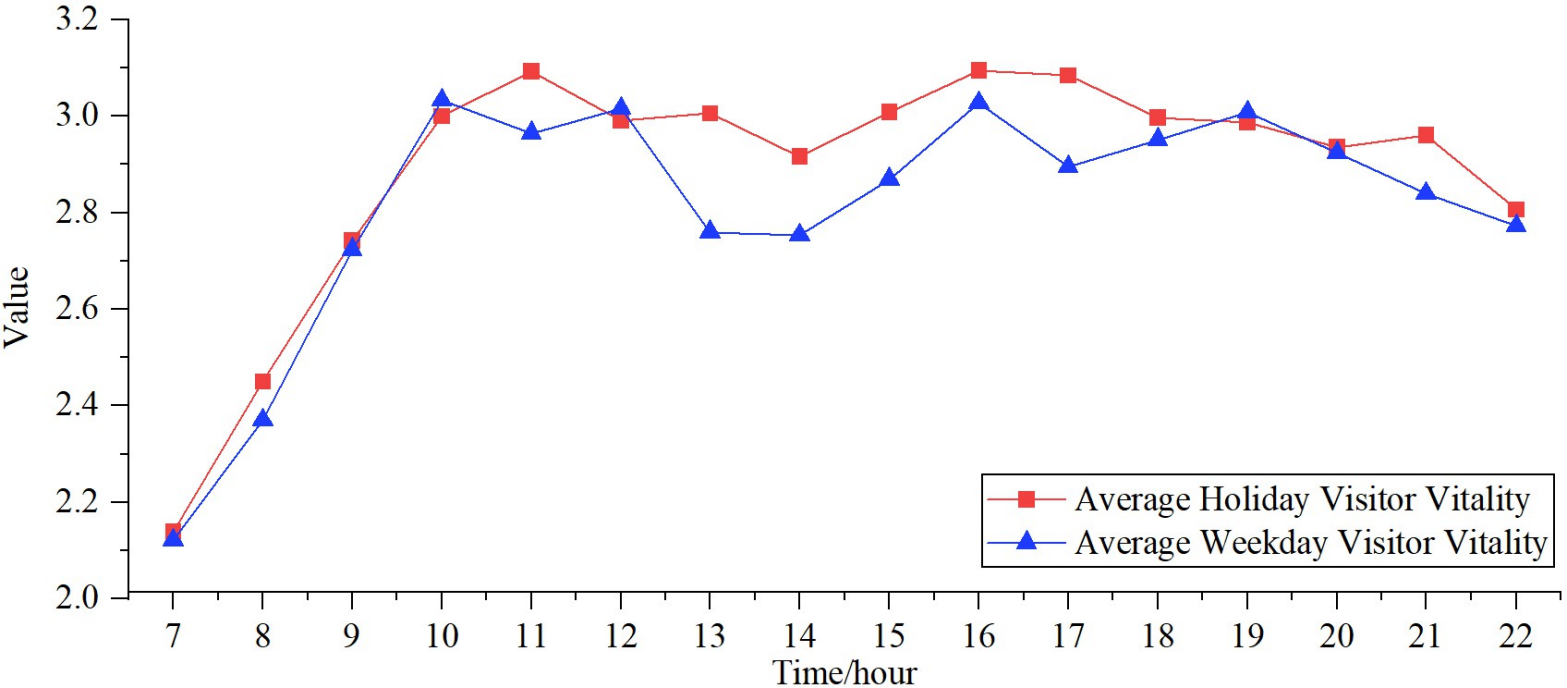
Temporal change in average visitor vitality.

The park visitor vitality value measures the intensity of visitors’ vitality during their visit. A higher value indicates a greater intensity of visitor vitality and a higher level of vitality in the park. This study aims to statistically determine the average thermal values of the park during the seven days of the National Day and the workweek. it analyses park visitors’ vitality levels on holidays and weekdays based on mean values (Fig 4). overall, there was a notable disparity in park usage between holidays and weekdays. During holidays, the vitality values of the park were consistently more outstanding than on weekdays. However, the difference in visitor vitality between peak hours on holidays and weekdays was less pronounced than during off-peak hours. The variations in vitality levels between 7:00–10:00 are similar on holidays and weekdays, with the slightest difference. It signifies that tourists favoured the time slot of 10:00 a.m. for engaging in activities at the park.

#### 3.1.1 Holiday park visitor vitality

The temporal trend of park visitor vitality on holidays reveals that the total park vitality exhibits a multi-peaked, undulating pattern with alternating periods of growth and decline. Two peak times occur at 11:00 and from 16:00 to 17:00. The initial surge occurs between 10:00 and 11:00, when individuals are actively participating in morning exercise routines. Furthermore, there are two consecutive declining patterns observed until 14:00 p.m, resulting in the formation of a trough. This is because there is a decrease in park vitality during that time period as visitors engage in mealtime and lunch breaks. Subsequently, the levels of vitality in the park progressively rise, resulting in a secondary peak of vitality for outdoor activities at 16:00. Eventually, the levels of vitality in the park start to gradually decrease, with a tiny rise observed between 20:00 and 21:00. This is because travelers have more leisure time during the holiday season, and they participate in evening fitness and walking activities.

#### 3.1.2 Weekday park visitor vitality

The vitality values of park visitors on weekdays exhibited considerable fluctuations, characterized by an overall M-shaped pattern. During the time period from 12:00 to 16:00, there is a more noticeable and distinct pattern of both downhill and upward "troughs". This aligns with the motivations behind individuals taking getaways or engaging in work during holidays. After 16:00, the park vitality exhibits an N-shaped pattern, with the average vitality level during the 19:00 time frame significantly surpassing that on holidays. This is due to the heightened inclination of the general population to engage in leisure and physical activities in parks upon completing their daily job obligations. Beyond, there is a pronounced decline in park vitality levels during weekdays.

### 3.2 Visitor vitality space differences

#### 3.2.1 Spatial distribution patterns

**Fig 5:**
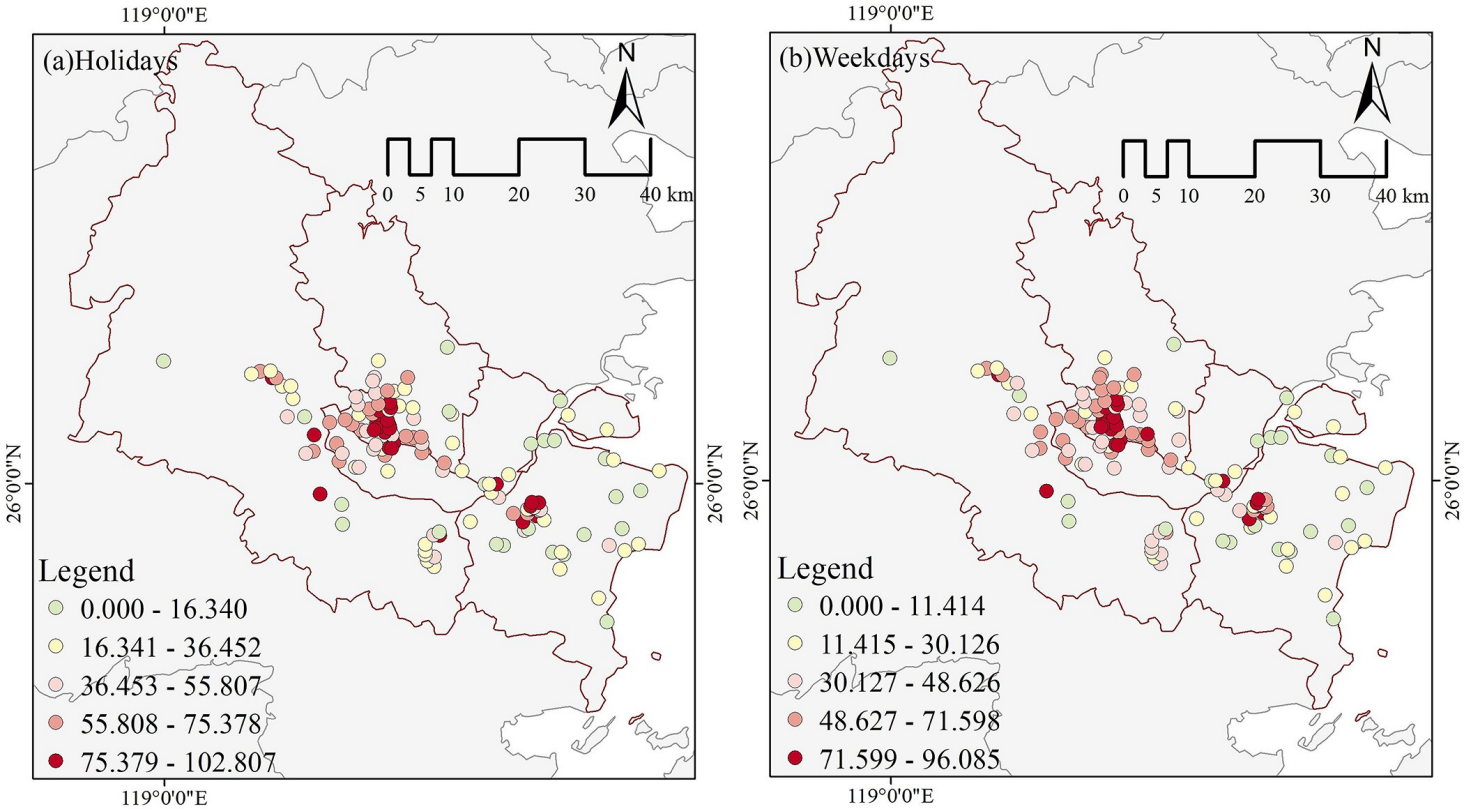
Spatial distribution patterns of visitor vitality.

The spatial character of the vitality of urban park visitors on holidays and weekdays was analyzed using ArcGIS 10.7. The analysis involved dividing the data into five gradients using the Jenks’ optimization (Fig 5). Overall, The spatial distribution of park visitors’ vitality on holidays and weekdays exhibits a pattern characterized by two central areas and a multi-chip. The two central areas of vitality districts are concentrated in the old city district (Gulou, Taijiang, and the northern section of Cangshan District) and the new city district (downtown Changle District). "multi-chip" low-vitality zones are characterized by an outward decline with a high-vitality core. Due to the prominent presence of the old parks in the old city district, which draws away potential tourists from Minhou County and the western portion of Jinan District that are next to the old city district, resulting in a decline in the park’s vitality rating. Although the new city district is located further away from the old city district, the park’s proximity to the seaside increases its worth of visit. Therefore, the levels of visitor vitality are elevated during the holiday season. Simultaneously, the vitality levels of parks on holidays and weekdays exhibit a "long-tail effect" characterized by a smaller number of highly vital parks and a more significant number of parks with low vitality. Meanwhile, there was no significant change in park tiers for holidays and weekdays.

#### 3.2.2 Spatial autocorrelation tests

A spatial autocorrelation analysis was conducted separately for visitor vitality in urban parks on holidays and weekdays. The findings indicated that the Moran’s *I* for vacations was 0.289 with a z-value of 10.963, whereas the Moran’s *I* for weekdays was 0.318 with a *z*-value of 12.041. The holiday and weekday visitor vigor are statistically significant at 1%. The data suggests that the visitor’s vitality to urban parks in the main urban area of Fuzhou is not randomly spread out spatially on holidays and weekdays; it shows a positively connected spatial distribution. High park vitality can have a positive correlation with the influence it has on nearby parks. Conversely, low vitality parks can have a negative impact on neighboring parks.

**Fig 6:**
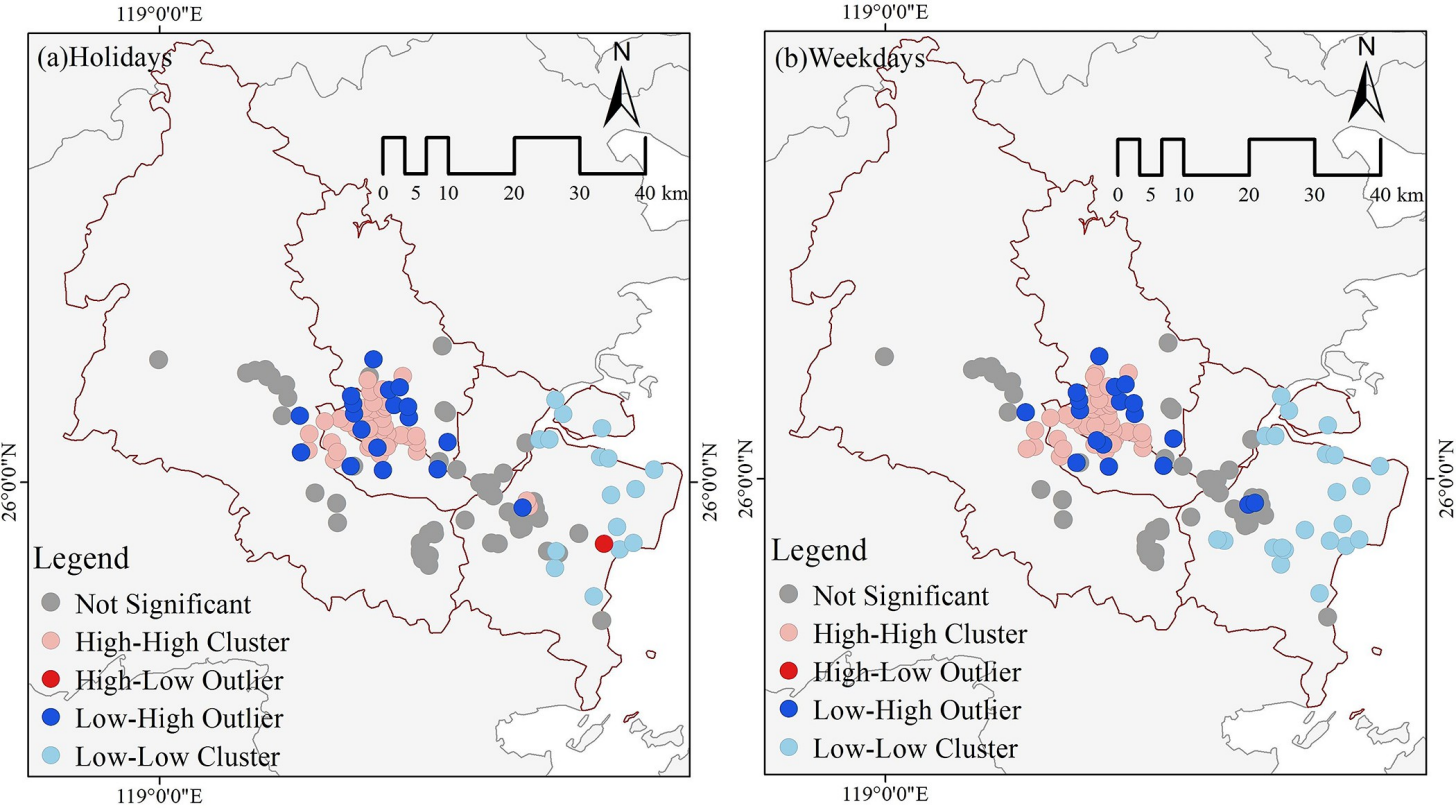
Spatial clustering characteristics of visitor vitality.

Fig 6 displays the outcomes of LISA spatial clustering for distinguishing holidays from weekdays. urban park visitors exhibited four distinct distributional clusters: High-High, High-Low, Low-High, and Low-Low (Fig 6A). During the holiday season, the visitor vitality of the parks are closely interconnected, with the overall boundaries of the Changeling District and nearby local governments serving as the limits. The "High-High" and "Low-High" zones are primarily located in the old city district. The space distribution of high-vitality parks in the old city district has a clustered pattern with significant variations in park vitality. In contrast, low-vitality parks are scattered around high-vitality parks. The "low-low" clustering area is located in Changle Mawei New city district. In this area, the general level of park visitor vitality is low, with only one park in the new city district having a high level of park visitor vitality, resulting in a "High-Low" clustering pattern. The LISA clustering analysis of the intensity of visitors on weekdays does not reveal any distinct "High-Low" clusters (Fig 6B), and the general separation is more noticeable. The "High-High" clusters are primarily located in the old city district, whereas the new city district is predominantly characterized by the presence of "Low-Low" clusters. This suggests a notable disparity in visitor vitality between weekends and weekdays in the new city district. Meanwhile, certain parks in the new city district exhibit higher levels of vitality during holidays.

### 3.3 Overall exploration of impact factors

#### 3.3.1 Overall characteristics

We employed vacation and weekday urban park visitor vitality values as the response variables while utilizing 25 impact indicators as the predictor variables. We applied the Natural breaks (Jenks) and Geometrical interval to systematically analyze and refine the indicators hierarchically. Meanwhile, we were utilizing geodetector to identify factors influencing visitor vitality in urban parks in the primary city of Fuzhou. Table 3 indicates that 15 indicators passed the significance level test on holidays, whereas 16 indicators passed the test on weekdays. The park’s vitality is primarily influenced by two primary factors: the features of the Peripheral location characteristics (G2) and the characteristics of the transportation environment and facilities (G3) (Table 3).

**Table 3:** Single factor detection results.

Type	variable	Weekday *q*-values	Weekday *p*-values	Holiday *q*-values	Holiday *p*-values
**Park attributes (G1)**	Park area (X1)	0.042	0.521	0.046	0.468
	Water body area (X2)	0.013	0.999	0.015	0.999
	Green Coverage (X3)	0.072	0.191	0.077	0.159
	Internal facilities (X4)	0.073	0.593	0.064	0.682
	Internal road network density (X5)	0.144	0.012 *	0.160	0.004 **
	Park building history score (X6)	0.149	0.655	0.145	0.653
	Average elevation (X7)	0.178	0.000**	0.169	0.000**
	Flat slope gradient (X8)	0.289	0.000**	0.262	0.000**
**Peripheral location characteristics (G2)**	Business Office Facilities (X9)	0.372	0.000**	0.344	0.000**
	Education and Medical Facilities (X10)	0.452	0.000**	0.434	0.000**
	Living Service Facilities (X11)	0.501	0.000**	0.491	0.000**
	Recreational Facilities (X12)	0.337	0.000**	0.314	0.000**
	Building Density (X13)	0.038	0.571	0.033	0.643
	Average building height (X14)	0.261	0.000**	0.257	0.000**
	Residential accommodation facilities (X15)	0.451	0.000**	0.428	0.000**
	Commercial shopping facilities (X16)	0.446	0.000**	0.448	0.000**
	Number of parking lots (X17)	0.010	0.999	0.011	0.999
**Characteristics of transportation infrastructure (G3)**	Road network density (X18)	0.244	0.000**	0.215	0.000**
	Average travel time cost (X19)	0.344	0.000**	0.331	0.000**
	Bus stop density (X20)	0.389	0.000**	0.371	0.000**
	Density of subway stations (X21)	0.177	0.037 *	0.165	0.058
	Park to Airport Time (X22)	0.091	0.032 *	0.074	0.070
	Park to Fuzhou Station Time (X23)	0.176	0.000**	0.192	0.000**
	Park to Fuzhou South Station Time (X24)	0.079	0.051	0.092	0.026 *
**Park Social Media Levels (G4)**	Park network attention (X25)	0.090	0.196	0.098	0.228

Note: "*" and "**" represent passing 5% and 1% significance tests, respectively.

#### 3.3.2 Park attributes (G1)

Urban park visitor vitality is only influenced by three variables in the park’s own features category: internal road network density (X5), average elevation (X7), and average slope (X8). Among these variables, X8 exhibits the most significant explanatory capacity regarding the influence of park vitality (0.289 on holidays and 0.262 on weekdays). It can be inferred that the inclination of the slopes in the park influences the decisions tourists make regarding their tours. During the holiday season, the impact coefficients of X7 and X8 decrease, suggesting that having a variety of park features for visitor transportation during this time will decrease the necessity for a single vertical landscape element. The holiday impact coefficient(X5), with a significance level of 0.160. This suggests that during the holiday period, tourists will be more concerned about the quality of the park’s road network system facilities and the roads’ diversity and level of interest. These factors will enhance the park’s tourist attraction during the holiday season.

#### 3.3.3 Peripheral location characteristics (G2)

The peripheral location characteristics group indicator is closely related to the life of citizens, reflecting the convenience, richness and reasonableness of the life around the city park, which naturally promotes the vitality of visitors most obviously. Seven of the nine indicators in the G2 group successfully passed the significance level test at a threshold of 0.01. The X11 facilities had the most significant impact coefficients on park vitality, with values of 0.501 and 0.491. It is essential to mention that the impact coefficients for weekdays for impact factors X9-X12, X14, and X15 are all greater than those for holidays. This suggests that tourists who travel during holidays are more focused on the park’s features or choose to go on outings and pay less attention to the development of the surrounding infrastructure. On holidays, X16 has a more significant impact than on weekdays, with a high coefficient of effect (0.488). On holidays, X16 has a more significant impact than on weekdays, with a high coefficient of effect (0.488). Commercial establishments like restaurants and shopping malls will likely attract tourists to the park.

#### 3.3.4 Characteristics of transportation infrastructure (G3)

The results of the single-factor analysis for the indicators in the transportation environment and facilities features category indicate that the impact factors X18-X20 and X23 all exhibit significant and high impact coefficients on park vitality. The findings suggest that the level of transportation accessibility has a direct impact on the organization of tourists’ itineraries and the overall happiness of their trip experience [34]. The X23 holiday coefficient is once again lower than on weekdays, indicating that travellers are more inclined to visit nearby parks on holidays. Furthermore, X22 fails to meet the criteria for statistical significance on holidays. This is because the airport is situated in the far suburb of Changle, and the cost-effectiveness of the high-speed train is superior to that of the airport. Consequently, a more significant number of tourists opt for high-speed rail travel. Although X24 Fuzhou South Station is a new high-speed rail station, it typically has lower tourist traffic than Fuzhou Station. Therefore, significant only during periods of high tourist traffic on holidays.

#### 3.3.5 Park social media levels (G4)

There was no significant correlation between internet attention (X25) and the vitality of urban park visitors, which deviates substantially from past scholarly research. Prior studies indicate that online attention is a preliminary indication of visitor vitality. Furthermore, heightened online attention towards urban parks can potentially lead to an increase in the number of tourists visiting them. This prejudice may stem from the atypical nature of Fuzhou as a tourist destination, resulting in a need for synchronization between its online and physical vibrancy.

#### 3.3.6 Two-factor interaction to detect sorting

**Fig 7:**
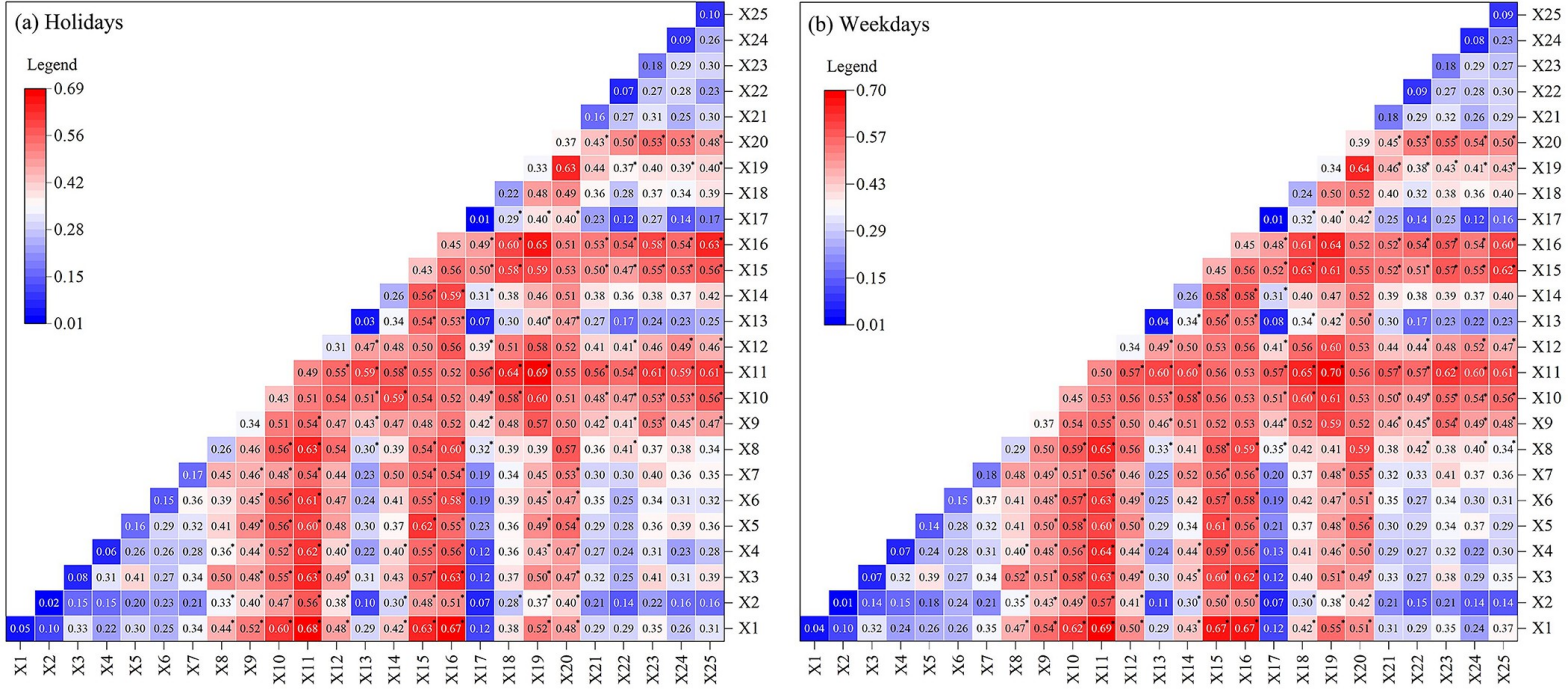
Two-factor interaction results. * is a two-way interaction passing a 0.05 significance test.

Single-factors analysis reveals essential factors in the spatial distribution of park vitality and quantifies the degree of these effects. However, interaction detection identifies interactions between different influences that are layered on top of each other to explain the impact of park vitality (Fig 7). The results indicate that the interaction between holidays and weekdays has two effect on park vitality, with both a two improtemnt and nonlinear enhancement. This suggests that combining these two factors significantly improves the ability to explain park vigour. The interactive explanatory power of the neighbourhood location characteristics (G2) dimension and the transportation environment and facilities characteristics (G3) dimension on weekdays and holidays were extreme (interaction value approximately greater than 0.5). This demonstrates that tourists consider a wide range of factors when planning their travels, including the availability of transportation facilities, the presence of fully equipped service facilities and other essential circumstances in the park’s surrounding area. Furthermore, in two-factor interaction, the X11 Living Service Facilities with the X1 park area on holidays and the X19 average commute time cost is highest on weekdays(0.69 and 0.70). The show tourists prioritize the availability of living service amenities when choosing a park during holidays. Still, on weekdays, they are more concerned about the cost of traveling to the park. This is also connected to the restricted duration of relaxation for tourists during weekdays. Nevertheless, it is essential to mention that the one-way test for X25 network attention did not pass the significance test. However, when the test was combined with the factors, the strength and significance of the effect were much more significant. This demonstrates that, apart from possessing a foundation of high-quality resources, the park must also use virtual communication channels and capitalize on traffic to enhance the park’s liveliness.

Among the elements that indirectly influence the outcome, there was a significant interaction between the average slope of X8 and factors within the G1 park’s characterisation dimension, with heat values of 0.4 or higher. Specifically, the interaction values associated with X3 green coverage are 0.50 and 0.52. The statement suggests that the presence of park landscape resources is a crucial foundation for satisfying the recreational requirements of tourists. The park’s abundant recreational amenities and establishing a high-quality ecological environment will give it an edge in enticing people to visit, as it will stimulate their Willingness to travel and acceptance for the park.

### 3.4 Analysis of spatial heterogeneity in impact factors

The study revealed that the spatial distribution of park vitality exhibited notable spatial clustering and heterogeneity. Moreover, the investigated parameters had varying degrees of impact on the park holiday and weekday effects coefficients.Hence, we conduct a more in-depth analysis of the spatial heterogeneity features of elements that influence the spatial vitality of holidays. This analysis can assist in formulating specific and optimum strategies to promote the vitality of distinct parks.

#### 3.4.1 Model selection and comparison

**Fig 8:**
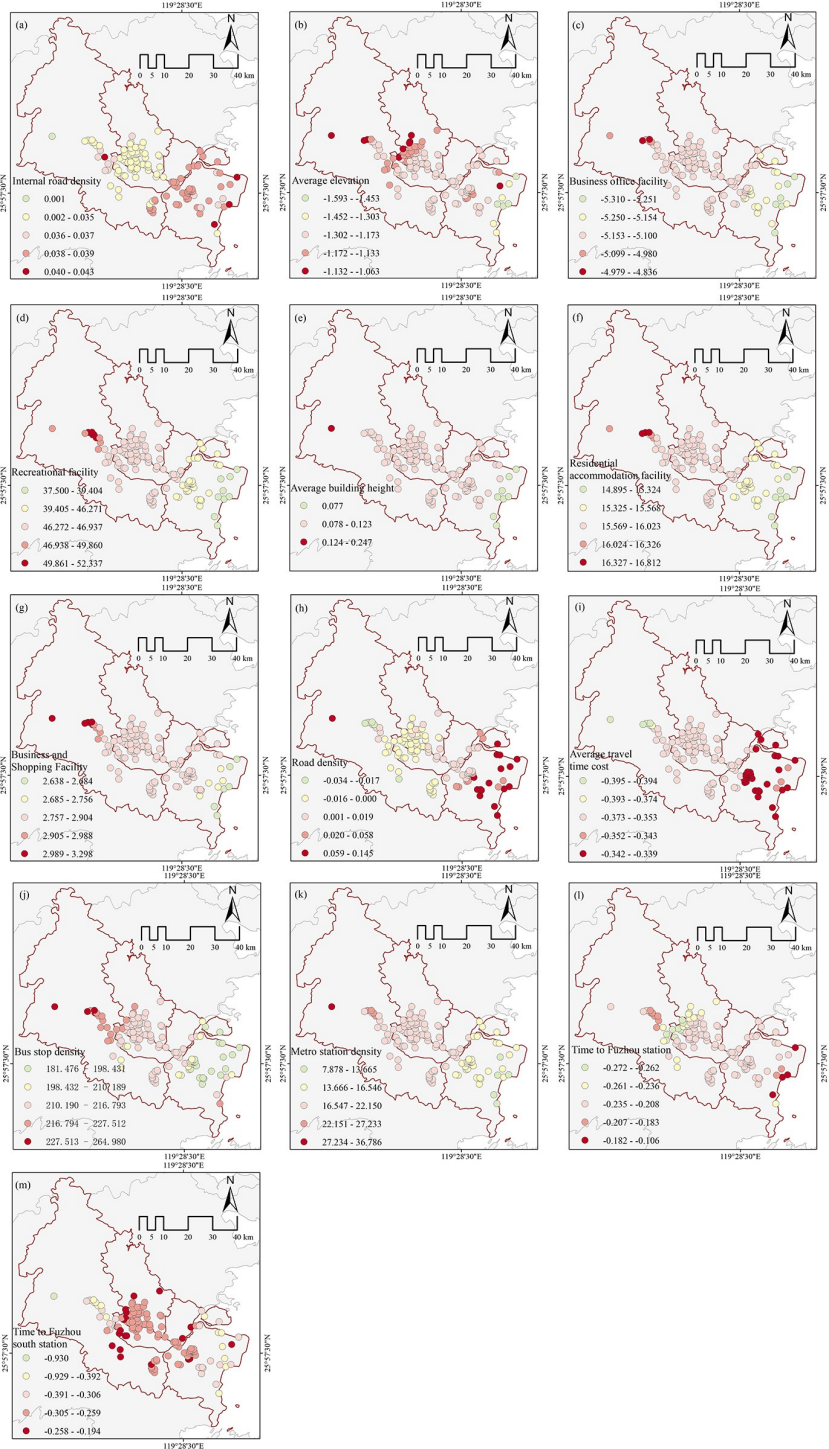
Spatial distribution of regression coefficients of the impact factor.

The initial step involved using the ordinary least square (OLS) method to identify the covariance of the influential factors that were deemed significant. Subsequently, the indicators with VIF (Variance Inflation Factor) values greater than 7.5 were eliminated in descending order, starting from the largest, to mitigate the presence of multiple covariances among the factors to some extent. Subsequently, the GWR and MGWR models were computed by amalgamating the values of the 13 influences that successfully met the multicollinearity and significance criteria, along with the dependent variable Holiday Vigor (Table 4). Upon analyzing the three groups of models, it is evident that the MGWR model exhibits the lowest AICc value (1165.09) and the highest adjusted R2 value (0.538). Overall, the run was improved and more well-suited. Thus, the MGWR model was employed in this study to examine the regional variability of visitor vigour and its affecting elements (Fig 8).

**Table 4:** Model index comparison.

Model indicators	OLS	GWR	MGWR
AICc	1671.48	1173.21	1165.09
R^2^	0.564	0.565	0.596
Adjusted R^2^	0.507	0.506	0.538

#### 3.4.2 Examining the arrangement of multi-scale spatially weighted regression coefficients

The spatial regression findings were shown using ArcGIS 10.7 software, which produced a spatial distribution map showing the regression coefficients of each influencing factor on the vitality of the holiday parks (Fig 8). Among these factors, the G1 dimension of the park’s own features has a low overall coefficient of influence on the density of the road network within X5 (0.001–0.043). The spatial distribution exhibits a progressive pattern from the old city district to the new city district. This indicates that the extensive road network within the parks in the new city district influences the travel decisions of visitors. This indicates that the availability of well-developed road infrastructure within the parks in the new city district influences the travel options of tourists. The average height X7 influence coefficient exhibits a negative correlation (Fig 8B), suggesting an inverse relationship between the park’s elevation and the visitor’s willingness to travel. The effect coefficient has a spatial distribution characterized by a gradual decline from the northwest to the southeast. This is influenced by the population density of the old city district and the park’s level of popularity.

The spatial distribution of impact coefficients within the G2 peripheral location characteristics all show an increasing trend from west to east (Fig 8D–8G). Among them, the influence coefficients of X12, X14-X16 are positively influenced and the level of X12 influence coefficient is higher(37-5-52.337). The impact indicators in this category include recreational activities, residential lodging, and commercial shopping and other open space service facilities. These facilities, due to their nature of providing opportunities for play, consumption, and relaxation, have the potential to attract a large number of citizens and contribute to increasing the overall vitality of the park. Simultaneously, the population density is greater in the old city district compared to the new city district, resulting in a more significant coefficient of influence on vitality. However, it is worth noting that the X9 commercial office amenity coefficient level shows a negative effect as (-5.310–4.836). Due to the fact that commercial offices largely cater to the public’s need for functional activities such as event management and office work, there is a low demand for park.

Characteristics of transportation infrastructure (G3), The impact coefficient of X18 road network density on park vitality exhibits a phenomenon of coexistence of positive and negative effects (Fig 8H). Negative correlation in the old city district(-0.034–0.000) and the new city district is positively correlation(0.00–0.145). The reason for this is that the old city district was planned and built earlier, resulting in a potentially disorganized and irrational road network. This indirectly impacts the willingness of tourists to visit. On the other hand, the new city district has a well-designed and convenient road network system, which encourages tourists to visit the parks in the new city district and enhances the overall visitor experience. X19 Fig 8I demonstrates a negative correlation between the average cost of travel time and the vitality of city park visitors. This suggests that the amount of time spent visiting parks is inversely connected to the number of people visiting parks. Additionally, lengthier travel time decreases people’s willingness to travel [35]. The bus stop density coefficient (Fig 8J) demonstrates a significant positive impact(181.476–264.980) and a spatial distribution pattern that increases from the southeast to the northwest (from the new city district to the old city district). This further indicates that convenient transit options enhance the inclination of tourists to visit [36,37]. Fig 8L and 8M shows that the influence coefficients of X23 on Fuzhou station time and X24 on Fuzhou south station time are inversely impacted. Nevertheless, the spatial distribution characteristics of the two coefficients differ dramatically. The X21 coefficient consistently increases from the northwest to the southeast, whereas the X22 coefficient displays the opposite direction.

## 4 Discussion

### 4.1 Park vitality hour characteristics

There is a notable disparity in the duration of park usage between holidays and weekdays, with all holiday park vitality ratings being consistently more significant than those on weekdays. The reason for this is that vacation visitors have more free time and are significantly more inclined to travel compared to weekdays.

Most visitors to the park occurred during the daytime, specifically at roughly 11:00 and 16:17:00, which aligns with the findings of prior studies [38,39]. This could be associated with variables such as climatic conditions and the presence of potential visitors. The parks department can manage and redirect the flow of visitors on park tours based on their travel preferences to guarantee a superior tour experience.

Regarding spatial distribution, high-vitality parks on holidays and weekdays are concentrated in the old city district. However, the distinction lies in that parks in the old city district experience a more significant influx of visitors’ vitality on holidays, resulting in a higher overall vitality rating. Nevertheless, high-vitality parks also draw away the vitality from parks in nearby areas, resulting in a geographical distribution that exhibits a "Low-High" clustering pattern. At the same time, part of the city park, due to the high quality and characteristics of tourism resources features to attract tourists from afar to play, such as Changle Binhai Park, which is the new city district most extensive coastal wetland park, seaside tours, sunny beaches and other attractions to attract holiday tourists gather [40], which formed the holiday Changle Binhai Park vitality and the formation of neighbouring parks, "High-Low"Space mismatch type. Hence, to address the potential disparities in spatial equity resulting from varying vitality levels in different parks, park management can implement a repositioning strategy highlighting lesser-known and distinctive attributes of low-vitality park areas. This strategy aims to evenly distribute visitor traffic by analyzing the characteristics of park resources and understanding visitors’ preferences.At the same time, the government should provide policy and endowment support for vibrant spaces in the lower zones to reduce the problem of equity in green space for citizens [41–43]. Furthermore, the radiative effect of high-vitality parks can still be observed for the "High-High" match type. Research has demonstrated that areas with high levels of vitality impact the focus and attention of the network in the nearby vicinity. This effect also extends to the vitality levels of visitors in parks. For instance, in the general development plan, it is advisable to construct park clusters with high vitality levels in established urban areas with stable visitors. This will help to enhance the consistent growth of visitor vitality in the parks. On the other hand, in the newly developed city district, the emphasis should be on creating parks of exceptional quality. This will stimulate the development of visitor vitality in these areas by establishing landmark parks that serve as representatives of the region’s progress.

### 4.2 The mechanism of the impact of park vitality

Previous research has identified several critical criteria influencing vitality in urban parks, including park size, water bodies, green coverage, and internal facilities [31,44,45]. However, our study did not find these factors to be statistically significant. Conversely, the density of the internal road network (X5), the average elevation (X7), and the average slope (X8) all passed the one-way test and demonstrated a positive connection. It demonstrates that the future design and development of parks should not solely focus on standardized factors like size and amount of green space. We can design more trails, corridors and add multi-dimensional experience content such as different heights of visual experience to meet the needs of tourists for high quality travel experience and diversified services, which in turn attracts tourists and enhances the attractiveness of urban parks.

The vitality of park visitors on holidays and weekdays is mainly influenced by the peripheral location features (G2) and the characteristics of transportation infrastructure (G3), as supported by numerous scholarly studies [46–48]. Indicates that urban parks are more vitality in regions with dense amenities and business establishments. Recreational and commercial service facilities enhance tourists’ trip experience by offering entertainment and food services, attracting people to gather in the region. The spatial distribution of Fuzhou City’s centres of gravity is concentrated in the old city district, resulting in a higher population density, better-developed road system, more affluent historical and cultural city parks, and more complete commercial retail venues compared to the new city district. This imbalance leads to a higher level of vitality and tourist engagement in the parks of the old city district. To attract visitors to new city district with low vitality parks (Changle Binhai District and Minhou High-tech Zone, etc.), we can enhance the surrounding infrastructure by increasing the availability of employment opportunities, commercial establishments, and educational facilities. This will encourage people to settle in these areas and ensure a consistent influx of visitors to the urban parks [49].

Furthermore, we observe a coexistence of both positive and negative effects of road network density on park life, a finding that contradicts a previous study conducted by Terry Hartig [50]. Hence, to determine the density of the road network surrounding the park, we must devise optimization algorithms based on the specific metropolitan region in which the park is situated. For example, in the old city district, we have to provide visitors with quick and easy access to the park tips to reduce visitors due to the intricacies of the road and reduce the satisfaction of the park. The new city district parks will speed up the construction of transportation roads and lines and peripheral transportation ancillary facilities (bus stops, subway stations, etc.) to provide visitors with various arrival routes to choose from.

We observed a noteworthy rise in the magnitude of the effect size after Two-factor interaction between the attention given to parks online and the characteristics of the surrounding location amenities (G2). This suggests that digitized park network promotion and locational resource advantages can increase park visitor vitality [51]. Therefore, on the basis of ensuring the improvement of service facilities, parks can be empowered by the traffic economy to establish a high-quality tourism brand, in order to promote the park’s external influence and dissemination effect. At the same time, combining with the tourism discipline that the network attention is the "precursor" of the attraction visitor vitality [52]. At the same time, park administrators can play a leading role in online network vitality, which can be used to monitor and warn the offline vitality of urban parks.

## 5 Conclusions

This study examines the temporal and spatial distribution patterns of visitor vitality in urban parks during National Day vacations and weekdays, using data on visitor vitality in urban parks located in the main urban area of Fuzhou. After that, by constructing a comprehensive indicator system, the influencing factors and local spatial heterogeneity relationships of visitor vitality in urban parks were explored using detectors and multi-scale geographically weighted regression models. This aids in precisely determining the spatial vitality status of parks, formulating distinct resource optimization techniques to direct park administration and planning, and successfully enticing visitors to enhance the expansion of urban park vitality. The results show that:(1) There is a notable disparity in the duration of park usage between holidays and weekdays, with parks being more appealing on holidays than on weekdays. Nevertheless, the highest levels of vitality occur specifically at 10:00 and 16:00. The overall vitality of the holiday park exhibited a multi-peaked, wavy pattern of increasing and decreasing. The "peak periods" occurred around 11:00, 16:00, and 21:00; The weekdays have a distinct M-shaped pattern, with the lowest point at 12:00. (2) The spatial distribution of visitors’ vitality on weekdays and holidays is characterized by a pattern of "two cores and multi-chip". The "two cores" are the high-vitality old city district, and the "multi-chip" are scattered in the new and old city district bordering and the newcity district in the city’s eastern part. There is a "long-tail effect" of fewer high-vitality parks and more low-vitality parks, and there is no significant change in park tiers between holidays and weekdays. (3) The peripheral location features (G2) and the characteristics of transportation infrastructure (G3) is a major factor in park vitality. The distinction lies in that the degree of the holiday element is smaller than that of the working day. Members of the general public are more likely to choose to visit parks located at a considerable distance and have insufficient amenities. The park qualities (G1) and social media levels (G4) showedshowed significant and heightened strength in their two-way interaction. The park qualities (G1) and social media levels (G4) showed significant and heightened strength in their two-way interaction. among others, The density of metro stations (X21) is only significant for the vitality of the Holiday Park. The distance to the airport (X22) is also essential for the vitality of the Holiday Park. This could be attributed to the fact that vacation travellers generate a substantial amount of outgoing traffic and strongly need various means of transportation. Various factors influence the vitality of holiday parks, and these characteristics have a significant regional variation. The positive indicator coefficients exhibit a spatial pattern characterized by a decline from the northwest to the southeast. The old city district displays more significant coefficients than the new one. Conversely, the negative indicators demonstrate the opposite trend. This may be related to the fact that holiday visitors have a high volume of outbound traffic and a high demand for all different transportation modes.

Due to the limited data collection of visitor vitality for only one National Day and one working week, the study’s accuracy is always inadequate. Nevertheless, this study represents a commendable effort to examine the level of visitor vitality in urban parks during holidays. We will persist in monitoring and acquiring Baidu heat map data and diversify our methods of gathering quantitative data on visitor vitality, such as incorporating noise and light data to quantify visitor engagement. This will enhance the credibility of our findings. Additionally, we will research visitor vitality’s spatial and temporal patterns across various holidays and workweeks.

## Supporting information

S1 File Appendices. (CSV)
