# Correction: A study of the changing characteristics and influencing factors of holiday visitor vitality in Urban parks: The case of Fuzhou, China

**DOI:** 10.1371/journal.pone.0345091

**Published:** 2026-03-17

**Authors:** Tingting Cui, Yongxiang Ye, Yingxin Zhuang, Qinlan Lin, Minlong Yan, Litian Zhang, Liying Zhu

The first author, Tingting Cui, is incorrectly noted as the corresponding author. The correct corresponding author is Liying Zhu. Liying Zhu’s email address is: zhuliying@fafu.edu.cn.

There are errors in the author affiliations. The correct affiliations are as follows:

Tingting Cui^1^, Yongxiang Ye^1^, Yingxin Zhuang^1^, Qinlan Lin^1^, Minlong Yan^1^, Litian Zhang^1^, Liying Zhu^1^

**1** Landscape Architecture and Art College, Fujian Agriculture and Forestry University, Fuzhou, Fujian, China
